# The “fat heat-up” phenotype: adipose tissue hypermetabolism on 18 F-FDG PET/CT predicts frailty in older patients with solid tumour*s*

**DOI:** 10.1007/s00259-026-07843-0

**Published:** 2026-03-21

**Authors:** Gursan Kaya, Serdar Ceylan, Meltem Gulhan Halil, Omer Ugur

**Affiliations:** 1 Department of Nuclear Medicine and Molecular Imaging, Turkish Ministry of Health, Yozgat City Hospital, Yozgat, Türkiye; 2 Faculty of Medicine, Department of Nuclear Medicine, Hacettepe University, Ankara, Türkiye; 3 Department of Geriatrics, Turkish Ministry of Health, Antalya City Hospital, Antalya, Türkiye; 4 Faculty of Medicine, Department of Geriatrics, Hacettepe University, Ankara, Türkiye

**Keywords:** Sarcopenia, F-18 FDG PET/CT, Frailty, Adipose tissue metabolism, Body composition, Deep learning, Opportunistic screening

## Abstract

**Purpose:**

Frailty in oncology is a major determinant of treatment toxicity and survival, and is often framed primarily as a muscle problem. Adipose tissue, however, is an active endocrine and metabolic organ, and its glycolytic activity on positron emission tomography/computed tomography with fluorodeoxyglucose (18 F-FDG PET/CT) may capture physiological vulnerability that is not reflected by body composition alone. We investigated the association between adipose glycolytic activity and frailty in older adults with solid tumours.

**Methods:**

We prospectively enrolled 104 adults (≥ 50 years) with solid malignancies (median age 63.5 years) who underwent clinical whole-body 18 F-FDG PET/CT and a comprehensive geriatric assessment on the same day. At the L3 level, adipose area and metabolic activity (SUVmean and rSUVmax; SUVp95-derived and reference-normalized hereafter referred as SUVmax for simplicity) were quantified using a deep learning segmentation pipeline (TotalSegmentator) with strict exclusion of visceral structures to isolate adipose signal. Frailty was assessed using the Clinical Frailty Scale (CFS) and the FRAIL Scale.

**Results:**

Total adipose area did not differ between frail and non-frail phenotypes (425.64 vs. 424.38 cm², *p* = 0.45). In contrast, adipose glycolytic activity was significantly higher in frail patients (SUVmean 0.30 vs. 0.20, *p* < 0.001). In multivariable logistic regression adjusted for age and sex, each 0.1-unit increase in adipose SUVmean was associated with 1.78-fold higher odds of frailty (95% CI 1.10–2.88, *p* = 0.002). Associations were directionally consistent across both frailty instruments.

**Conclusion:**

Adipose hypermetabolism on 18 F-FDG PET/CT, despite low absolute SUV values, appears to track frailty independently of adipose quantity, supporting a “fat heat-up” phenotype as a marker of diminished physiological reserve. Routine oncologic PET/CT may therefore provide an opportunistic, imaging-derived frailty signal that can precede overt morphological deterioration in body composition.

**Significance Statement:**

Frailty is a high-impact and frequently under-recognised driver of treatment intolerance and adverse outcomes in oncology, yet comprehensive geriatric assessment remains resource-intensive and inconsistently implemented. In this prospective cohort, adipose tissue was quantified using a fully automated Total Segmentator-based pipeline, providing an objective and reproducible measure of adipose FDG uptake at the L3 level. This single quantitative feature, already embedded in routine 18F-FDG PET/CT, aligned with frailty across two independent frailty scales and multiple geriatric assessment domains, independent of age and sex. If externally validated, adipose FDG uptake could function as an opportunistic imaging-derived frailty flag to trigger earlier geriatric input, support treatment individualisation, and enable physiologic risk stratification in trials and real-world practice.

**Supplementary Information:**

The online version contains supplementary material available at 10.1007/s00259-026-07843-0.

## Introduction

Body composition, particularly skeletal muscle mass, adipose tissue distribution and tissue metabolic activity, has emerged as an important determinant of prognosis, treatment tolerance and quality of life in patients with cancer. Of these components, sarcopenia has been consistently linked to increased treatment toxicity, postoperative complications, functional decline and mortality in oncology patients [1]. However, in addition to muscle wasting, changes in the amount, distribution and metabolic behaviour of adipose tissue may also play a key role. Adipose tissue is now recognised as a dynamic endocrine organ that produces a variety of adipokines, inflammatory mediators and metabolically active substances. Such pathways facilitate close interaction between adipose and muscle tissue, contributing to systemic inflammation, metabolic dysregulation and age-related physiological decline. Thus, these mechanisms provide a biological basis for the development of frailty [2–4].

Frailty describes a state of reduced physiological reserve and heightened vulnerability to external stressors, resulting in increased risks of falls, hospitalisation, functional dependence, and mortality [5]. Although traditionally studied in older adults, frailty is increasingly acknowledged as a crucial consideration in oncology, where the burden of cancer and its treatments can accelerate physiological deterioration beyond that expected with ageing alone [6]. There is a growing body of evidence that suggests abnormalities in body composition, including increased visceral adipose, reduced subcutaneous adipose, intramuscular adipose infiltration and unfavourable muscle-to-adipose ratios, are associated with frailty and adverse clinical outcomes [7, 8]. In oncology, sarcopenia combined adipose tissue quantity has been associated with reduced survival [9].

Positron emission tomography/computed tomography (PET/CT) with fluorodeoxyglucose (FDG) provides a unique opportunity to quantify not only the volume but also the metabolic activity of adipose tissue through standardised uptake values (SUVs). Previous studies have explored the prognostic relevance of adipose tissue metabolic activity across various malignancies, revealing associations with survival and treatment response [10–12]. Nevertheless, the relationship between adipose tissue SUVs and frailty has not been systematically investigated, and the potential of adipose metabolic markers to indicate physiological vulnerability remains unclear.

Given the clinical importance of frailty in cancer care and the established use of FDG PET/CT in oncological assessment, examining the relationship between adipose tissue SUVs and frailty may offer valuable insights into the metabolic determinants of vulnerability. Such biomarkers may support improved risk stratification, guide personalised treatment planning, and facilitate the early identification of high-risk individuals. The aim of this study is therefore to investigate the relationship between FDG PET/CT-derived adipose tissue SUV parameters and frailty in older patients with solid organ malignancy, and to explore the potential of adipose glycolytic activity as a biomarker of frailty.

## Materials and methods

### Study design and population

**Fig. 1:**
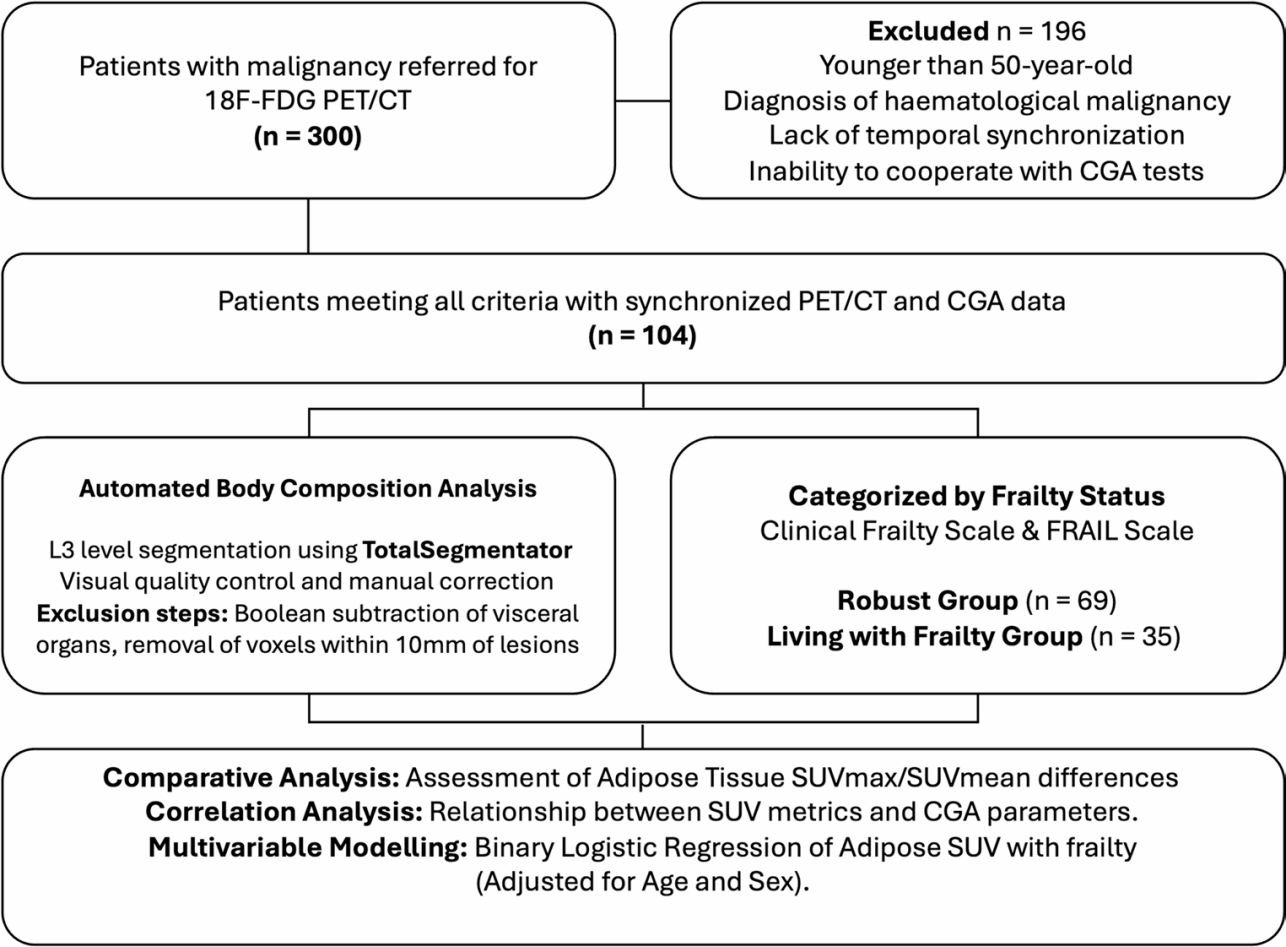
Flow chart of the study design

The study was performed prospectively in a university hospital with participants meeting the specified inclusion criteria. Inclusion criteria were: aged 50 or older, having a solid organ malignancy, performing a FDG PET/CT scan on the day of the comprehensive geriatric assessment (CGA) and being able to cooperate with the tests. Participants who could not cooperate with the tests and whose PET/CT could not be performed on the CGA day for any reason were excluded. A brief overview of the study design is provided in Fig. 1.

This research was conducted as a prospective observational cohort study with a statistical analysis plan developed a priori. To minimize bias and avoid retrospective data mining, all endpoints, variables of interest, and testing procedures were defined before data acquisition began. The primary endpoint was to evaluate the association between FDG PET/CT-derived adipose metabolic parameters (SUVmean and rSUVmax) and frailty status, defined using both the Clinical Frailty Scale and the FRAIL scale. Secondary endpoints included correlations between adipose metabolic markers and (i) individual domains of the CGA and (ii) objective physical performance metrics; these secondary analyses were interpreted as supportive/exploratory within the pre-specified framework.

Each patient underwent a CGA which was performed by a geriatrician on the day of PET/CT. The following were noted: age, sex, weight, height, education, marital status, chronic diseases, medications, falls, activities of daily living, nutritional status, frailty status, handgrip strength, 4-metre gait speed, and timed up-and-go test. Multimorbidity is used to describe the coexistence of two or more chronic diseases [13]. The term polypharmacy refers to the daily administration of a minimum of five medications [14].

PET/CT indications were determined by the oncologist who followed the patient and the nuclear medicine physician who would perform the procedure. No additional images were obtained for the study; measurements were made from standard images.

The subjects’ weight and height were recorded in the absence of footwear and wearing light attire. A hand-held dynamometer (T.K.K.5401; Takei Scientific Instruments, Tokyo, Japan) was used to measure handgrip strength. We asked patients to walk four metres at their normal pace to measure their walking speed using a digital watch. A gait speed of less than 0.8 m per second indicates poor physical performance [15].

### Frailty assessment

Frailty was assessed using two scales: Clinical Frailty Scale (CFS) and Frail Scale.

The development of CFS was by Rockwood et al., with participation by patients in the Canadian Study of Health and Aging. CFS is based on clinical judgment by the physician. It assigns a score between one and nine. This is based on activity, function, and disability. Its application does not require the use of tools or the running of laboratory tests. Scores of 5 or more are categorised as ‘living with frailty’ [16]. The validity and reliability of CFS in the Turkish context has been demonstrated by Ozsurekci et al. [17]. There are studies showing that CFS has been used in patients aged 50 and older [18].

FRAIL scale is comprised of five domains. The Geriatric Advisory Panel of the International Academy of Nutrition and Ageing developed it as an easy-to-use, time-efficient frailty scale for use by all healthcare professionals. No tools or tests are required to complete it. The frailty of the patient is determined by questioning them about fatigue, resistance, ambulation, illnesses and weight loss. A score of three or more is considered to be frail [19]. Turkish reliability and validity were addressed by Hymabaccus et al. [20]. The FRAIL scale has been used in patients aged 50 and over [21].

### PET/CT protocol

#### Imaging protocol and data acquisition

In this cohort, we analysed quantitative body composition and metabolic PET parameters derived from total body segmentations in a consecutive series of adult patients who underwent 18 F-FDG PET/CT. All examinations were performed on a PET/CT system (Discovery IQ Gen2; 4 ring system) using a standardised protocol. Patients fasted for at least 6 h before imaging, had a blood glucose level below 150 mg/dL at injection and received 3–5 MBq/kg 18 F-FDG intravenously. PET acquisition commenced approximately 60 min post injection and was performed for 2 min per bed position (5–6 bed positions).

Images were reconstructed using an ordered subset expectation maximisation algorithm with point spread function modelling (matrix 192 × 192). A low dose CT scan (120 kV, automatic tube current modulation) was acquired for attenuation correction and anatomical localisation and reconstructed with 3 mm slice thickness.

#### Automated body composition analysis

Body composition segmentation was performed using TotalSegmentator [22], an open-source deep learning pipeline for fully automated three-dimensional organ and tissue annotation. Processing was carried out on a high-performance GPU server to enable volumetric extraction of multiple muscular and adipose compartments. All automated segmentations were visually reviewed by an experienced nuclear medicine physician, with manual corrections applied where necessary. TotalSegmentator runs open-source and can be implemented routine workstations for the research purposes. Even with basic computers that have only CPU, fast processing completes under 5 min for total body segmentation of low-dose CT images and it is validated for body composition analysis externally [23].

**Fig. 2:**
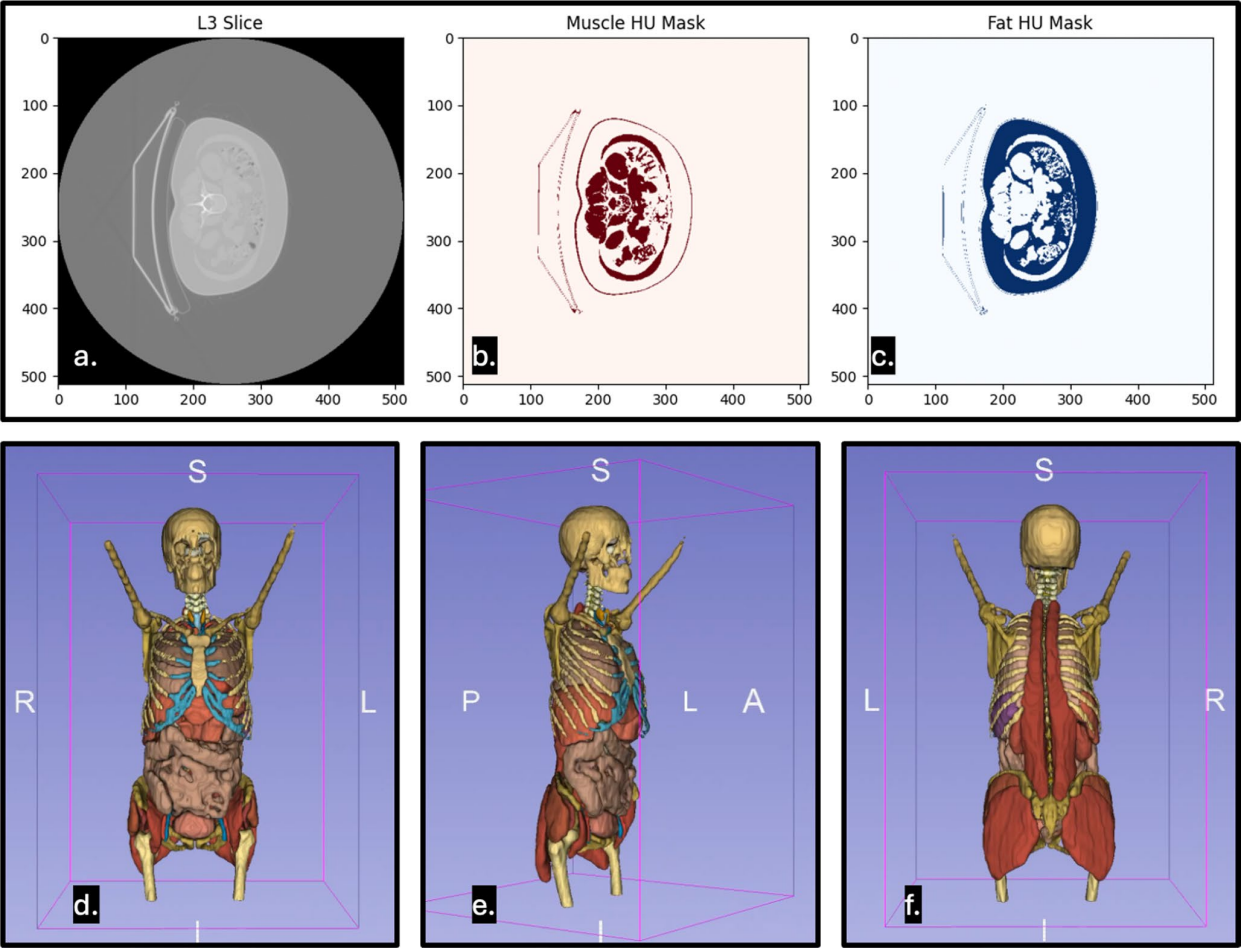
Segmentation workflow for body composition analysis.(**a-c**) Cross-sectional analysis at the third lumbar vertebra (L3) level. (**a**) Unprocessed axial CT image. (**b**) Skeletal muscle (and soft tissue) segmentation mask (red overlay) generated by TotalSegmentator. (**c**) Adipose tissue segmentation mask (blue overlay) differentiating subcutaneous and visceral fat compartments. (**d-f**) Three-dimensional (3D) volumetric renderings derived from the total-body CT dataset. The anterior (**d**), lateral-oblique (**e**), and posterior (**f**) views illustrate the automated segmentation of skeletal muscle, skeleton, and internal organs in a whole-body field of view

Adipose tissue was segmented on the CT component generating separate masks for visceral and subcutaneous fat. The L3 level was defined as the axial slice intersecting the midpoint of the third lumbar vertebral body on CT. Because PET and CT were acquired in the same session on an integrated PET/CT system, no additional registration beyond the vendor’s standard attenuation-correction alignment was required. To reduce partial-volume and spill-in effects, adipose masks were eroded by 1–2 voxels in-plane, and voxels adjacent to non-adipose structures with potential physiologic uptake (bowel, urinary tract, major vessels, liver and spleen) were excluded using Boolean subtraction of the corresponding organ masks. In addition, all voxels within 10 mm of any metabolically active tumour lesion (defined visually on PET/CT) were removed to minimise tumour-adjacent spillover. Adipose uptake was summarised as SUVmean within the final VOI, and SUVmax was operationalised as the 95th percentile of the voxel-wise SUV distribution (SUVp95 referred as robust (r) SUVmax) rather than the single highest voxel, in order to improve robustness to image noise. An example output from the automated segmentation process is shown in Fig. 2. Additional subgroup analyses were performed according to metabolic muscle activity and fat–muscle distribution indices [24]. Muscle-related indices were included as supportive body-composition checks rather than primary outcomes. CT-derived skeletal muscle measures, including erector spinae–based metrics, were used to compare muscle mass proxies between frail and robust groups to evaluate whether differential muscle wasting could plausibly inflate SUV-based adipose uptake estimates.

### Statistical analysis

The data was analysed using the Statistical Package for the Social Sciences (SPSS) 24.0. The expression of categorical variables was as numbers and percentages. As there were no normally distributed continuous variables in the data, these were expressed as median and interquartile range between 25th-75th percentiles (IQR). Comparisons of continuous variables were made using the Mann-Whitney U-test since these variables are not normally distributed, and for categorical values, the chi-squared test was used. Spearman rho was used in the correlation analysis. Binary logistic regression analysis was performed to indicate that the relationship between frailty and adipose tissue SUV values ​​was independent of age and sex. A two-tailed p-value of 0.05 or lower was considered to be statistically significant.

## Results

The median (IQR) age of the 104 patients included in the study was 63.5 (58.0–72.0) years, and 51.9% (n = 54) of them were female. Sixty-eight (65.4%) patients were overweight or obese. 26.9% (n = 28) of participants were living with multimorbidity, and 22.1% (n = 23) had polypharmacy. The most common cancer type was breast cancer (n = 34, 32.7%), followed by lung cancer (n = 26, 25.0%), and gastrointestinal system tumours (n = 17, 16.3%). According to both the CFS and FRAIL scale, 35 patients (33.7%) were living with frailty. The median (IQR) adipose tissue rSUVmax was 0.4 (0.3–0.5), and the median (IQR) adipose tissue SUVmean was 0.3 (0.2–0.3) (Table 1).

**Table 1:** Demographic, clinical and imaging characteristics (*N* = 104)

Age* (years)	*N* (%)
	63.5 (58.0–72.0) *(51.0–81.0)*
Sex (female)	54 (51.9)
Body Mass Index* (kilogram per square meter)	26.4 (23.4–30.6) *(16.7–42.2)*
Normal	36 (34.6)
Overweight	41 (39.4)
Obese	27 (26.0)
Marital Status (not married)	16 (15.4)
Education (≤ 5 years)	37 (35.6)
Smoker	59 (56.7)
Surgery	70 (67.3)
Multimorbidity (≥ 2 chronic diseases)	28 (26.9)
Polypharmacy (≥ 5 medicines)	23 (22.1)
Medicine Count*	2.0 (1.0–4.0) *(0.0–13.0)*
Malignancy Type	
Breast	34 (32.7)
Lung	26 (25.0)
Gastrointestinal	17 (16.3)
Others	21 (20.2)
More than one primary	6 (5.8)
Chemotherapy	66 (63.5)
Radiotherapy	29 (27.9)
Compherensive Geriatric Assessment	
Katz Activities of Daily Living	6.0 (5.0–6.0)
Instrumental Activities of Daily Living	6.0 (5.0–8.0)
Mini-Nutritional Assessment Short Form	13.0 (10.0–14.0) *(5.0–14.0)*
At Risk of Malnutrition/Malnourished (≤ 11)	35 (33.7)
Clinical Frailty Scale*	4.0 (4.0–5.0) *(3.0–9.0)*
Living with Frailty (≥ 5)	35 (33.7)
FRAIL Scale*	2.0 (1.0–3.0) *(0.0–4.0)*
Living with Frailty (≥ 3)	35 (33.7)
SARC-F*	2.0 (0.5-4.0) *(0.0–9.0)*
At Risk of Sarcopenia (≥ 4)	29 (27.9)
History of Falling	14 (13.5)
4-meter Gait Speed* (meter per second)	0.99 (0.75–1.05) *(0.40–1.40)*
Slow (≤ 0.8 m per second)	27 (26.0)
Handgrip Strength* (kilogram)	
Female (*n* = 54)	19.4 (14.6–22.6) *(10.3–32.1)*
Male (*n* = 50)	28.4 (21.6–32.3) *(8.4–45.4)*
Timed Up & Go Test* (second)	10.5 (9.2–14.3) *(7.1–30.0)*
Imaging (At level Third Lumbar Vertebra)	
Total Adipose Tissue Area* (square centimeter)	425.01 (294.69-537.26) *(62.30-837.72)*
Adipose Tissue rSUVmax*	0.4 (0.3–0.5) *(0.2–0.8)*
Adipose Tissue SUVmean*	0.3 (0.2–0.3) *(0.1–0.7)*

*N *Number, *median (IQR) *(minimum-maximum)*, *SUV* Standardized Uptake Value

Higher rSUVmax and SUVmean values were observed in patients living with frailty. According to CFS, p-value was 0.002 for rSUVmax and p < 0.001 for SUVmean. As per the FRAIL scale, p = 0.01 for rSUVmax and p = 0.002 for SUVmean (Table 2 ).

**Table 2:** Adipose tissue imaging results based on frailty status (N=104)

	Robust(n=69, 66.3%)	Living with Frailty(n=35, 33.7%)	*p*
Clinical Frailty Scale			
Total Area* (squarecentimeter)	424.4(337.6-518.3)	425.6(243.9-543.8)	0.45
rSUVmax*	0.3 (0.3-0.4)	0.4 (0.35-0.5)	0.002
SUVmean*	0.2 (0.2-0.3)	0.3 (0.3-0.4)	<0.001
FRAIL Scale			
Total Area* (squarecentimeter)	422.7(312.8-518.3)	435.6(243.9-543.7)	0.81
rSUVmax*	0.3 (0.3-0.4)	0.4 (0.3-0.5)	0.01
SUVmean*	0.2 (0.2-0.3)	0.3 (0.2-0.4)	0.002

*N*, Number, *median (IQR), *SUV*, Standardized Uptake Value

rSUVmax was statistically significantly correlated with all CGA parameters except Katz Activities of Daily Living (ADL) and handgrip strength, and SUVmean was significantly correlated with all CGA parameters except handgrip strength. rSUVmax and SUVmean were positively correlated with CFS (CC: 0.30, p = 0.002 for SUVmax & CC: 0.34, p < 0.001 for SUVmean) and FRAIL Scale (CC: 0.32, p = 0.001 for SUVmean & CC: 0.39, p < 0.001) (Table 3 ). The relationship between adipose glycolytic activity and frailty scales is further illustrated in Supplementary Figs. 1 and 3; these scatter and box plots provide a transparent view of data distribution, confirming the upward trend of SUV metrics in frail individuals despite the expected overlap in low-range values.

**Table 3:** Correlation between comprehensive geriatric assessment parameters and adipose tissue imaging findings (*N* = 104)

	rSUVmax		SUVmean	
	CC	*p*	CC	*p*
Clinical Frailty Scale	0.30	**0.002**	0.34	**< 0.001**
FRAIL Scale	0.32	**0.001**	0.39	**< 0.001**
Katz Activities of Daily Living	-0.18	0.07	-0.20	**0.04**
Instrumental Activities of Daily Living	-0.32	**0.001**	-0.35	**< 0.001**
Mini-Nutritional Assessment Short Form	-0.25	**0.01**	-0.33	**0.001**
SARC-F	0.30	**0.002**	0.33	**0.001**
4-meter Gait Speed	-0.33	**0.001**	-0.32	**0.001**
Handgrip Strength	-0.17	0.08	-0.17	0.08
Timed Up & Go Test	0.33	**0.001**	0.36	**< 0.001**

*N* Number, *SUV *Standardized Uptake Value, *CC *Correlation Coeffcient

To facilitate clinical interpretability, we performed a supplementary ROC analysis evaluating adipose SUVmean and rSUVmax for discriminating frailty. For CFS-defined frailty (CFS ≥ 5), adipose SUVmean achieved an AUC of 0.694 (95% CI 0.584–0.804) with an optimal threshold of 0.25 (sensitivity 77.1%, specificity 59.4%). For FRAIL-defined frailty, the corresponding AUC was 0.669 (95% CI 0.554–0.784) with the same SUVmean threshold of 0.25 (sensitivity 71.4%, specificity 56.5%). Full diagnostic performance metrics, including PPV and NPV for both frailty definitions and rSUVmax, are provided in Supplementary Table 1. These thresholds should be interpreted as exploratory and require external validation before being considered actionable clinical cut-offs.

The results of binary logistic regression analysis using frailty status as the dependent variable are presented in Table 4. According to CFS, a 0.1-unit increase in adipose rSUVmax increased the probability of living with frailty by 1.66-fold (95% CI 1.10–2.50, p = 0.02), independent of age and sex. Similarly, a 0.1-unit increase in adipose SUVmean increased the probability of living with frailty by 1.78-fold (95% CI 1.10–2.88, p = 0.002), independent of age and sex. According to FRAIL scale, a 0.1-unit increase in adipose rSUVmax increased the probability of living with frailty by 1.53-fold (95% CI 1.05–2.25, p = 0.03) and a 0.1-unit increase in adipose SUVmean increased the probability of living with frailty by 1.71-fold (95% CI 1.08–2.71, p = 0.02), independent of age and sex. In age and sex-adjusted models, higher adipose FDG uptake remained associated with frailty. Given the observational design and the potential for residual confounding, these findings should be interpreted as hypothesis-generating rather than establishing adipose FDG uptake as an independent biomarker.

**Table 4:** Regression analysis of the relationship between frailty andimaging results

	Odds ratio	95% Confidenceinterval	*P*
Clinical Frailty Scale			
rSUVmax (For a 0.1 UnitChange)	1.66	1.10-2.50	0.02
SUVmean (For a 0.1 UnitChange)	1.78	1.10-2.88	0.002
FRAIL Scale			
rSUVmax (For a 0.1 UnitChange)	1.53	1.05-2.25	0.03
SUVmean (For a 0.1 UnitChange)	1.71	1.08-2.71	0.02

The models were created by defining frailty status as the dependentvariable and including age, sex, and one of the imaging findings (a total of 4 different models). The reason for setting the independent variable to 3 isto prevent the models from overfitting, given that the number of patientsliving with frailty is 35

## Discussion

The present study demonstrates a significant association between adipose tissue glycolytic activity, quantified by FDG PET/CT-derived SUV parameters, and frailty in older adults with solid organ malignancies. Frailty, assessed using both CFS and Frail Scale, consistently correlated with increased adipose SUVs. Our findings suggest that metabolic activation of adipose depots may reflect systemic physiological vulnerability, supporting the emerging concept of adipose tissue as an active endocrine and inflammatory organ rather than a passive energy store.

Much of the existing literature on frailty and body composition has focused on muscle mass and muscle quality, as well as radiologically derived adipose quantity and distribution. These factors are primarily assessed via CT. For example, a study involving a large sample size and using CT scans at the L3 vertebral level found that a higher visceral adipose tissue (VAT) area and a lower skeletal muscle density were independently associated with frailty, at least in men [7]. Similarly, the infiltration of adipose into muscle tissue and the deposition of adipose in abnormal locations have been associated with decreased muscle strength and poorer physical performance — two key features of frailty [25, 26].

Conversely, sarcopenic obesity has emerged as a high-risk phenotype for frailty and adverse outcomes in older adults. A recent meta-analysis showed that older adults with sarcopenic obesity were at significantly greater risk of frailty than robust individuals [27]. Furthermore, among community-dwelling older adults, individuals with sarcopenic obesity were found to have significantly higher odds of pre-frailty and frailty compared to those with sarcopenia or obesity alone [28]. Collectively, these data support a model in which both muscle wasting and deterioration in muscle quality, as well as abnormal adipose accumulation and distribution, contribute to frailty. However, most previous studies have only assessed morphometric parameters, such as tissue volume or area, adipose-to-muscle ratios, and attenuation. The metabolic activity of adipose tissue, which may reflect processes such as inflammation, lipolysis, lipid turnover or metabolic reprogramming, has rarely been considered in the context of frailty.

Indeed, there is growing evidence in oncologic imaging that the metabolism of adipose tissue on FDG PET/CT may have prognostic significance. For example, in patients with pancreatic adenocarcinoma, high FDG uptake in visceral adipose tissue has been associated with poorer overall survival, regardless of tumour stage and other confounding factors [11]. Similarly, altered adipose tissue metabolic activity has been proposed as a potential biomarker for cancer-associated cachexia [29]. The present study builds on these observations by suggesting that metabolic activity in adipose tissue may also indicate systemic vulnerability, as defined by frailty — particularly in solid cancer patients, for whom cachexia, inflammation and metabolic dysregulation are common. Thus, the present findings introduce a new dimension by showing that the metabolic behaviour of adipose tissue, as well as its quantity or distribution, may be important for frailty. This could have practical implications. FDG PET/CT imaging, which has an established role on staging and treatment planning in many cancer patients, could be opportunistically used to assess frailty risk without the need for additional imaging or invasive tests.

There are biologically plausible mechanisms through which increased metabolic activity in adipose tissue could be related to frailty. Adipose tissue is metabolically active and secretes various cytokines and adipokines, which modulate systemic inflammation, insulin sensitivity, muscle protein turnover and anabolic signalling. These processes are implicated in muscle wasting, myosteatosis and reduced muscle function [30]. Altered adipose metabolism may reflect or promote ectopic lipid deposition in muscle, impairing muscle quality and function even if muscle mass is preserved [31]. In cancer patients, tumour metabolic activity, systemic inflammation, and metabolic derangements may lead to changes in adipose tissue behavior — increased lipolysis, altered glucose uptake, adipose redistribution — which in turn may accelerate frailty or physiological decline. Observations in cachexia suggest that abnormal adipose glucose uptake is associated with poorer outcomes [32, 33].

One plausible mechanism for the association between higher adipose SUV and frailty is the role of adipose tissue as a maladaptive “glucose sink”. In oncologic settings, systemic inflammation and tumour-derived mediators can drive a phenotypic switch of white adipose tissue toward browning or broader metabolic reprogramming [34]. In this hypermetabolic state, adipose depots sequester disproportionate amounts of glucose from the circulation and channel it into thermogenesis rather than long-term storage, resulting in futile energy expenditure [35]. This “nutrient steal” may deprive skeletal muscle of critical substrates, promoting accelerated muscle catabolism and functional decline even before sarcopenia becomes radiologically apparent [36, 37]. The observation of elevated adipose glycolytic activity in frail patients in our cohort is consistent with this model and supports the concept that adipose tissue behaves not as a passive reservoir, but as an active metabolic competitor that answers to the systemic inflammation and contributes to systemic energy depletion [38].

Although absolute SUV values in adipose tissue were low, the potential clinical impact resides in the total metabolic burden rather than in focal intensity. Adipose tissue constitutes a substantial volumetric compartment, so even a modest increase in glycolytic activity per unit volume may translate into a substantial shift in whole-body energy expenditure [39]. A transition from a near-quiescent baseline (for example SUV around 0.1) to a persistently low-grade active state (SUV around 0.4) represents a several-fold increase in glucose utilisation across this extensive reservoir. Physiological modelling indicates that although the specific resting metabolic rate of adipose tissue (approximately 4.5 kcal/kg/day) is intrinsically low compared with skeletal muscle (approximately 13 kcal/kg/day) or highly active visceral organs (approximately 440 kcal/kg/day), its sheer volume makes it clinically relevant. When this relatively modest per-kilogram expenditure is scaled to an average 20-kg adipose compartment and globally upregulated, the resulting cumulative demand constitutes a substantial metabolic burden for the whole organism [40]. Viewed in aggregate, this “mass effect” may act as a chronic systemic energy drain and could contribute more meaningfully to the physiological depletion seen in frailty than focal metabolic metrics alone would suggest. Importantly, the ROC-derived thresholds (e.g., SUVmean ≥ 0.25) are intended to enhance clinical interpretability within a low-uptake range and should not be viewed as definitive diagnostic limits without external validation. In real-world scenarios, however, these values may serve as opportunistic imaging-derived flags to trigger formal geriatric assessment and personalized risk stratification.

 A key methodological concern in frailty research is whether higher adipose SUV could reflect a normalization artifact rather than true compartment-specific uptake differences, particularly if frail patients have lower lean mass. Although liver uptake is sometimes used as an internal reference, hepatic SUV can vary in older oncology populations due to steatosis/fatty infiltration and treatment-related effects, which may limit its usefulness as a control in this setting [41]. We therefore addressed this issue more directly using CT-derived body composition measures from the same PET/CT examinations. Skeletal muscle metrics, including erector spinae–based measures, were comparable (typically *p* > 0.1) between frail and robust groups, which makes a systematic inflation of SUV due to differential muscle wasting less likely. In addition, our adipose quantification was VOI-based (segmentation-defined adipose compartments) and included a noise-robust rSUVmax operationalized as SUVp95 rather than a single-voxel SUVmax, further reducing susceptibility to image noise in the low-uptake range. Taken together, these considerations suggest that the observed “Fat Heat-Up” signal is unlikely to be driven primarily by normalization artifacts. 

Several CT-based indices at the L3 level have been associated with frailty and adverse outcomes in prior work. In our cohort, however, CT-derived adipose morphology metrics at L3 (for example, adipose area and related distribution indices) were comparable between robust and frail participants, suggesting limited discriminatory value of a CT-only approach in this specific setting. By contrast, adipose FDG uptake differed between groups, supporting the concept that metabolic alterations may provide complementary information that is not captured by morphology alone. One plausible interpretation is that FDG uptake reflects earlier or more dynamic adipose tissue dysfunction and systemic stress responses that precede measurable changes in adipose quantity or attenuation on CT. Future multi-center studies should directly compare CT-only versus combined CT + PET models and evaluate whether metabolic adipose phenotyping improves risk stratification and clinical decision-making beyond established CT-derived indices [7–9].

The fact that CFS and Frail Scale both showed similar associations with adipose SUV strengthens the internal validity of the results. Although these instruments capture different aspects of frailty—CFS relying on clinician judgement [16] and Frail Scale representing a phenotype-based construct [19]—the consistent relationships suggest that adipose metabolic dysregulation reflects a shared biological substrate underlying various frailty constructs. This convergence supports the potential integration of metabolic imaging biomarkers into frailty assessment frameworks, which may be particularly valuable in oncology where complex interactions between cancer burden, treatment toxicity and host physiology complicate clinical evaluation.

All 18 F-FDG PET/CT examinations in this cohort were performed with a non-contrast, low-dose CT for attenuation correction. This choice reduces protocol-related variability in quantitative uptake measures and provides a consistent substrate for body composition analysis. In contrast-enhanced acquisitions, iodinated contrast can affect attenuation maps and produce modest shifts in measured SUVs, with the magnitude depending on contrast timing, vascular enhancement, and the attenuation-correction implementation [42]. From a sarcopenia and adipose quantification perspective, non-contrast CT is also preferable because it preserves stable HU-based tissue thresholds that underpin automated segmentation. Contrast can transiently change apparent tissue density, which may blur muscle–fat boundaries and introduce additional measurement variability [43]. Restricting our analysis to non-contrast CT therefore strengthens the internal validity of the “Fat Heat-Up” phenotype by reducing the likelihood that the observed adipose uptake differences are driven by contrast-related attenuation effects. The generalizability of these findings to contrast-enhanced protocols should be evaluated in future studies.

The present study has several limitations. Firstly, the cross-sectional design precludes causal inferences; we cannot determine whether increased adipose SUV precedes frailty, vice versa, or if they are both consequences of underlying disease processes (e.g. cancer, inflammation or cachexia). Secondly, frailty is a complex, multidimensional syndrome and the method of assessing frailty used may not capture all the relevant aspects. Furthermore, FDG PET/CT-derived metrics may correlate differently with different frailty definitions. Thirdly, although we adjusted for major confounders such as age, BMI, comorbidities and cancer stage, residual confounding factors such as inflammation, nutritional status and activity level cannot be excluded. Fourthly, and importantly, most prior evidence linking adipose tissue metabolism to clinical outcomes originates from survival/prognosis studies (e.g. cachexia, mortality) rather than functional vulnerability, so translation to frailty requires caution. Finally, our findings may not be generalisable: our sample consisted of cancer patients who underwent FDG PET/CT for clinical reasons, and therefore may not represent broader oncological populations or non-cancerous older adults.

On the other hand the present study merits several methodological strengths that distinguish it from existing work. First, the prospective design and strict synchronisation of imaging with clinical assessment provide a high degree of temporal reliability. Performing the CGA and FDG PET/CT on the same day minimises bias arising from fluctuating physiological states, a common limitation in retrospective cohorts with variable time intervals. Second, the use of TotalSegmentator, a fully automated deep learning-based pipeline, ensured reproducible and observer-independent imaging metrics, overcoming the variability inherent in manual segmentation that characterises much of the earlier body composition literature. Third, internal validity is strengthened by the parallel application of two conceptually distinct frailty instruments: the clinician-judgement-based Clinical Frailty Scale and the phenotype-oriented FRAIL Scale. The consistent association between adipose tissue hypermetabolism and frailty across both tools, independent of age and sex, supports the robustness and biological plausibility of our findings. Finally, the close collaboration between nuclear medicine and geriatrics enabled an integrated assessment of metabolic, functional and body composition parameters, providing a more comprehensive view of the patient’s physiological reserve than imaging or clinical assessment alone could offer.

## Conclusion

Given the routine use of FDG PET/CT in oncology, integrating adipose tissue SUV analysis offers a cost-effective and non-invasive opportunity to identify patients at high risk of frailty, treatment intolerance, and functional decline. Identifying high-risk patients could inform decision-making, for example by tailoring treatment intensity, providing closer monitoring, offering early supportive care; such as nutrition and physical therapy, or implementing frailty-targeted interventions. Our results support the idea that metabolic activity in adipose tissue can be used as a biomarker of physiological reserve and vulnerability. This could complement traditional frailty assessments, such as clinical scales, muscle mass and functional tests. Longitudinal studies are needed to assess whether high adipose tissue uptake of FDG predicts the onset of future frailty, functional decline, morbidity or mortality. Intervention studies might explore whether modifying adipose metabolic activity affects the trajectory of frailty. Adipose FDG uptake on routine oncologic PET/CT may provide an opportunistic signal of systemic vulnerability; however, clinical implementation will require external validation, prespecified thresholds, and assessment of incremental value beyond age, comorbidity, and performance measures.

## Supplementary information

Below is the link to the electronic supplementary material.

Supplementary material 1

## Data Availability

The datasets generated during and/or analysed during the current study are available from the corresponding author on reasonable request.
