# Supplementary material for: The “fat heat-up” phenotype: adipose tissue hypermetabolism on 18 F-FDG PET/CT predicts frailty in older patients with solid tumours
Source: Eur J Nucl Med Mol Imaging. 2026 Mar 21;53(8):5022–32. doi: 10.1007/s00259-026-07843-0 (PMC13249925; doi:10.1007/s00259-026-07843-0)
Supplement: Supplementary file 1 — Supplementary material 1 [file 259_2026_7843_MOESM1_ESM.docx]

Supplementary Material


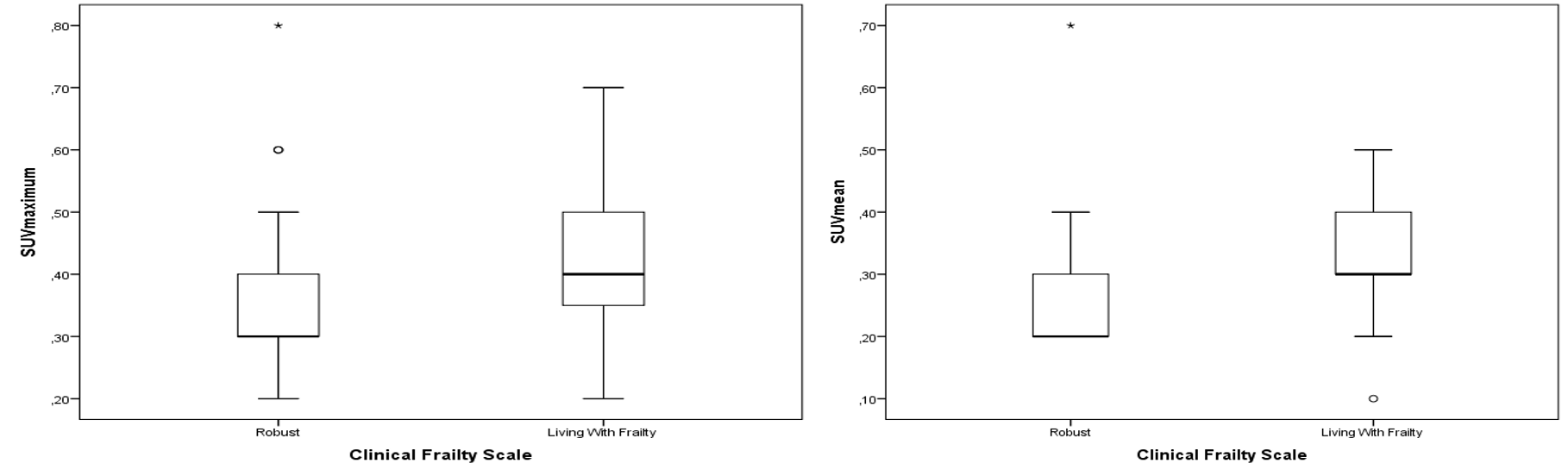


**Figure 1.** Distribution of SUVs (SUVmaximum as rSUVmax95p and SUVmean as mean of L3 vertebrae level adipose tissue area) between robust and frail groups according to Clinical Frailty Scale


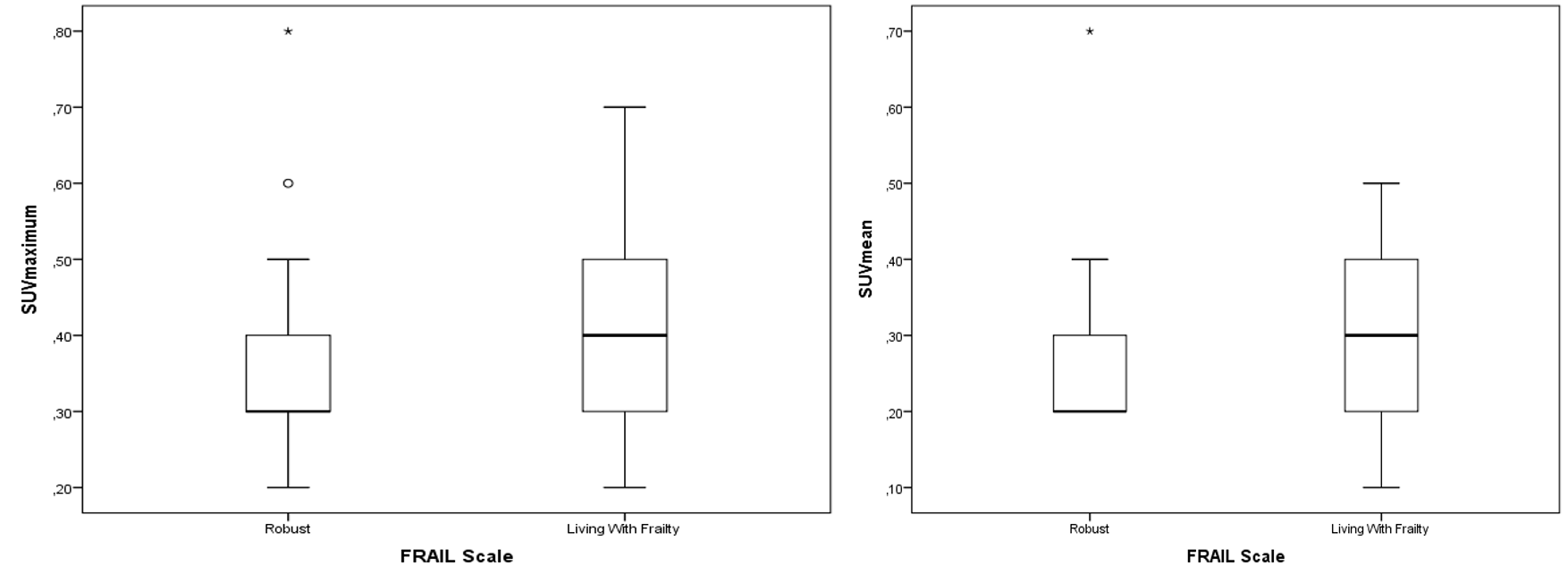


**Figure 2.** Distribution of adipose tissue SUVs (SUVmaximum as rSUVmax95p and SUVmean as mean of L3 vertebrae level adipose tissue area) between robust and frail groups according to FRAIL Scale


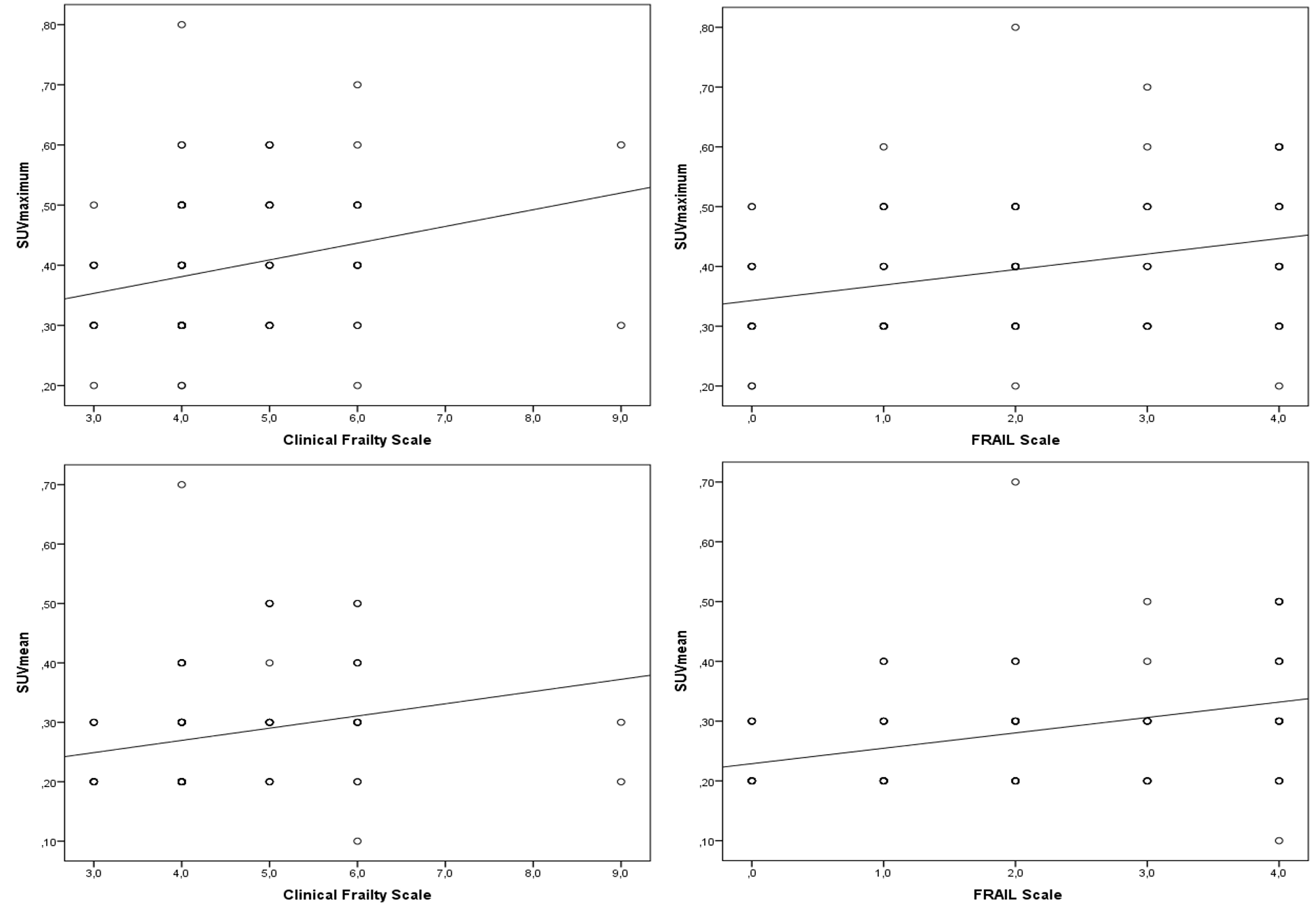


**Figure 3.** Scatter plots for adipose tissue SUVs (SUVmaximum as rSUVmax95p and SUVmean as mean of L3 vertebrae level adipose tissue area) and Clinical Frailty Scale-FRAIL Scale

**Table 1.** ROC analysis and diagnostic performance of optimal SUV thresholds

|  | **Cut-off Value** | **Area Under Curve** | **95% Confidence Interval** | **p** | **Sensitivity (%)** | **Specificity (%)** | **Positive Predictive Value (%)** | **Negative Predictive Value (%)** |
| --- | --- | --- | --- | --- | --- | --- | --- | --- |
| **Clinical Frailty Scale** | | | | | | | | |
| SUVmax | 0.35 | 0.681 | 0.570-0.792 | 0.003 | 74.3 | 55.1 | 45.6 | 80.9 |
| SUVmean | 0.25 | 0.694 | 0.584-0.804 | 0.001 | 77.1 | 59.4 | 49.1 | 83.7 |
| **FRAIL Scale** | | | | | | | | |
| SUVmax | 0.45 | 0.648 | 0.532-0.764 | 0.01 | 42.9 | 78.3 | 50.0 | 73.0 |
| SUVmean | 0.25 | 0.669 | 0.554-0.784 | 0.005 | 71.4 | 56.5 | 45.5 | 79.6 |
| SUVmax defined as adipose tissue rSUVmax95p and SUVmean defined as mean SUV of L3-vertebrae level adipose tissue area. | | | | | | | | |
